# Optimization of isolation and cultivation of bacterial endophytes through addition of plant extract to nutrient media

**DOI:** 10.1111/1751-7915.12291

**Published:** 2015-05-20

**Authors:** N Eevers, M Gielen, A Sánchez-López, S Jaspers, J C White, J Vangronsveld, N Weyens

**Affiliations:** 1 Centre for Environmental Sciences, Hasselt UniversityAgoralaan Building D, Diepenbeek, 3590, Belgium; 2 Edafología, Soil and Environmental Chemistry Lab, Colegio de Postgraduadoskm 36.5 Carretera Mexico-Texcoco, Estado de Mexico, 56230, Mexico; 3 Interuniversity Institute for Biostatistics and Statistical Bioinformatics, Hasselt UniversityAgoralaan Building D, Diepenbeek, 3590, Belgium; 4 Department of Analytical Chemistry, Connecticut Agricultural Experiment Station123 Huntington Street, New Haven, CT, 06511, USA

## Abstract

Many endophytes have beneficial effects on plants and can be exploited in biotechnological applications. Studies hypothesize that only 0.001–1% of all plant-associated bacteria are cultivable. Moreover, even after successful isolations, many endophytic bacteria often show reduced regrowth capacity. This research aimed to optimize isolation processes and culturing these bacteria afterwards. We compared several minimal and complex media in a screening. Beside the media themselves, two gelling agents and adding plant extract to media were investigated to enhance the number and diversity of endophytes as well as the growth capacity when regrown after isolation.

In this work, 869 medium delivered the highest numbers of cultivable bacteria, as well as the highest diversity. When comparing gelling agents, no differences were observed in the numbers of bacteria. Adding plant extract to the media lead to a slight increase in diversity. However, when adding plant extract to improve the regrowth capacity, sharp increases of viable bacteria occurred in both rich and minimal media.

## Introduction

Plant-associated bacteria, both endophytic and rhizospheric, are known to have beneficial effects on the host plant. These plant growth-promoting relationships can be further exploited to achieve improved growth of food and feed crops (Taule *et al*., 2012), higher biomass production of energy crops (Weyens *et al*., 2015) and greater tolerance for growth on marginal lands (Ker *et al*., 2012). Furthermore, many bacteria possess an inherent capacity to cope with contaminants, which may make them suitable for improving phytoremediation efficiency (Fester *et al*., 2014; Weyens *et al*., 2015). Rhizospheric bacteria are known to be more easily cultivable than comparable endophytic species (Bafana, 2012). Endophytes reside within plant tissues and are therefore exposed to a more specific and stable habitat than rhizospheric bacteria. However, endophytes are also more closely associated with, and likely dependent on, the plant than are rhizospheric bacterial populations. Between endophytes, a distinction can be made between obligate endophytes (Croes *et al*., 2013) and facultative endophytes (Kamnev *et al*., 2005). Obligate endophytes are expected to be the hardest to cultivate, since they require more specific conditions and are therefore the main focus of this manuscript.

To thoroughly investigate endophytic bacterial communities and to potentially exploit their beneficial effects by means of inoculation, the organisms must be cultivable under laboratory conditions. Organism isolation is the first critical step in this cultivation procedure. A common endophyte isolation protocol consists of a surface sterilization of the plant tissues followed by crushing the plant tissues and plating the slurry onto nutrient medium. Sterilization protocols have been previously optimized in our laboratory for many plant species, including the use of sterilizing agents such as sodium hypochlorite, ethanol and hydrogen peroxide, as well as the optimal conditions for their application (Barac *et al*., 2004). Specifically, sterilization is achieved by submersing the plant tissues into a predetermined concentration of sodium hypochlorite for 1–5 min, varying with identity of the plant species and the organ of interest. Afterwards, the plant tissues are rinsed several times in sterilized distilled water, with the final water being plated onto undiluted rich medium to verify sterility. An efficient surface sterilization results in high amounts of endophytic growth on agar plates which indicates that there is no damage from the sterilization to the endophytic population, while there is minimal bacterial growth in the last surface rinsing water. However, the nutrient medium that is used subsequent to sterilization can also have a major influence on isolation efficiency. Many different media are mentioned in literature, depending on the research goal and species used. In general, for the isolation of plant-associated bacteria, a distinction can be made between complex, rich media that contain high amounts of somewhat undetermined nutrients and minimal media that contain significantly lower yet precise amounts of nutrients. Although the choice of a suitable growth medium is crucial during isolation, a comparative study of different nutrient media types has not been published. The nutrient medium will obviously affect both the number and diversity of endophytes that can be isolated from a specific plant tissue and it may also determine the ultimate cultivability of some endophytic bacterial strains altogether. In spite of a growing and robust literature on endophytes, cultivation-dependent techniques still strongly underestimate the number of bacteria present in plant tissues. The literature generally suggests that only 0.001% to 1% of the endophytes present in plant tissues are cultivable (Torsvik and Øvreås, 2002; Alain and Querellou, 2009). In addition, we have observed that many of the endophytic strains that grow during the initial isolation cannot actually be recultivated under laboratory conditions. We speculate that this may be due to the presence of residual plant-specific compounds and metabolites that are still present during the isolation but are not present during eventual recultivation. In fact, it should not be surprising that ‘crushed’ plant tissue contains compounds not present in synthetic bacterial growth media, but that are ultimately necessary for the growth of endophytic species.

Based on a literature review, we selected and compared different bacterial nutrient media, as well as different gelling agents to solidify the medium. After the optimal medium for isolation was identified, the addition of plant extract was tested as an amendment to promote the regrowth of previously isolated endophytes. In the original isolations, endophytes were isolated from *Cucurbita pepo* plants that were grown in vermiculite. Vermiculite itself contains low numbers of bacteria and is therefore a suitable substrate to grow plants when focusing on obligate endophytes. For the regrowth experiment, previously isolated endophytes from root, shoot and seed tissue of *Arabidopsis thaliana* and seed endophytes of *Crotalaria pumila* that showed difficulties growing after the original isolation were tested.

Two types of growth media were selected: complex and minimal media. These media contain all elements considered important for the non-selective growth of bacteria. A complex medium contains water, carbon sources, salts and a source of amino acids from fungal, plant or animal origin (yeast extract, tryptone, peptone, etc.). These media are called undefined because the exact composition of the amino acid source is not known (Madigan *et al*., 2014). Three types of undefined media were selected: Trypticase Soy Agar (TSA), Casein-Starch and 869 medium (1/10 diluted). Secondly, three minimal, defined media were selected: 284+ C, M3 and M9. Complex and minimal media might lead to different yields of cultivable endophytes because of the different growth conditions provided by the media. Complex media contain high amounts of nutrients such as sugars and amino acids, which implies that most (cultivable) bacterial species can grow easily on these plates. However, fast-growing, dominant bacterial strains might overgrow slow-growing endophytic strains, whereby the latter do not get the opportunity to develop. Minimal media, on the other hand, provide a strict amount of nutrients, which leads to a slower and more selective growth, but might also allow slow growing endophytic strains a chance to develop. Furthermore, the composition of minimal media might mimic the *in planta* conditions better than the rich, complex media, and therefore might allow an easier adaptation for endophytes (Alain and Querellou, 2009).

To utilize these media in Petri dishes, a gelling agent is required. Agar has been used for over 100 years as the general gelling agent in microbiology (Tamaki *et al*., 2009). However, it might be possible that certain bacterial strains are restricted in their growth by exposure to agar. More recently, gellan gum has been proposed as a possible alternative gelling agent (Tamaki *et al*., 2005). Gellan gum is a bacterial polysaccharide produced by *Sphingomonas* species (Sa-Correia *et al*., 2002). Gellan gum has been used for plant tissue culture (Cavallaro *et al*., 2014), human tissue culture (Gantar *et al*., 2014) and culturing of water and soil bacteria (Stott *et al*., 2008). However, a comparison between agar and gellan gum has not been previously reported for endophytic bacterial strains. Therefore, their functionality was investigated and compared in this study.

## Results and discussion

### Optimization of the isolation of endophytes

#### Screening for different bacterial nutrient media

**Fig 1:**
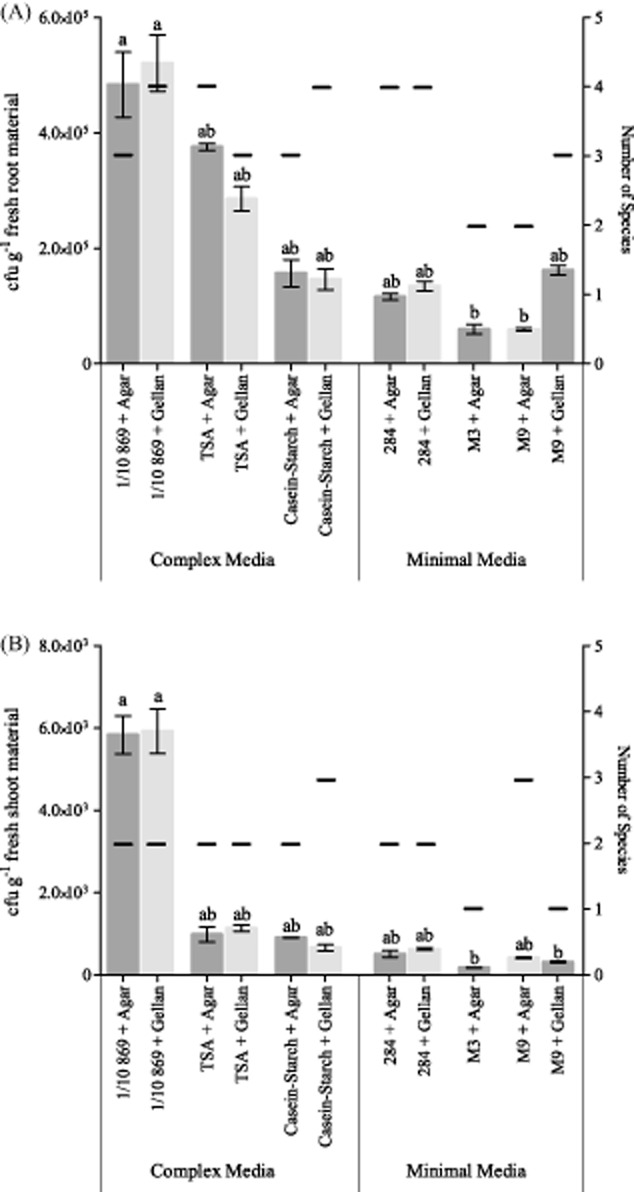
Comparison of the number of bacteria (in colony-forming units (cfu) per gram of fresh plant material) growing on different bacterial growth media with both agar and gellan gum as gelling agent (average of three technical repeats). The number of species that were visually distinguishable are given on the secondary y-axis. No data are available for M3 + Gellan. Bars with different letters are significantly different from each other (*P* < 0.05).A. Bacteria isolated from root material.B. Bacteria isolated from shoot material.

The nutrient media that are used during endophyte isolation will strongly influence the number and diversity of cultivable bacteria. The isolates from mixed root/shoot samples of three *C. pepo* plants were plated on six different media (Table 1) and after 5 days of incubation, the numbers of colony-forming units (cfu g^−1^) and species were determined (Fig. 1). In agreement with the literature, root tissue contained approximately 100-fold more bacteria, as well as visually distinguishable species, than did the shoot material (Gutiérrez-Ginés *et al*., 2014; Shehzadi *et al*., 2014).

**Table 1:** Composition of the bacterial growth media

	1/10 869 (Mergeay *et al*., 1985)	TSA (McCullough, 1949)	Casein-Starch (Wellington and Cross, 1983)	284+ C (Schlegel *et al*., 1961)	M9 (Sambrook and Russell, 2001)	M3 (Dedysh *et al*., 1998)
CaCl_2_.2H_2_0	0.035			0.030	0.010	
CaCO_3_			0.020			0.020
Casein			0.300			
CoCl_2_				190 × 10^−6^		
CuCl_2_				17.0 × 10^−6^		
Cyclohexemide						0.050
Fe(III)NH_4_ Citrate				4.80 × 10^−3^		
FeSO_4_.7H_2_0			0.010			200 × 10^−6^
Fructose				0.540		
Gluconate				0.660		
Glucose				0.520	4.00	
Glucose D+	0.100					
H_3_BO_3_				62.0 × 10^−6^		
KCl				1.490		
KH_2_PO_4_			2.00		3.00	0.466
KNO_3_			2.00			0.100
Lactate				0.350		
MgCl_2_.6H_2_0				0.200		
MgSO_4_.7H_2_0			0.050		0.490	0.100
MnCl_2_				100 × 10^−6^		
MnSO_4_.4H_2_0						20.0 × 10^−6^
Na_2_HP0_4_.2H_2_0				0.040	12.8	0.732
Na_2_SO_4_				0.430		
NaCl	0.500	5.00	2.00	4.68	0.500	0.290
NaMoO_4_				36.0 × 10^−6^		
NH_4_Cl				1.07	1.00	
NiCl_2_				24.0 × 10^−6^		
Sodium proprionate						0.200
Soytone		5.00				
Starch			10.0			
Succinate				0.810		
Thiamine.HCl						0.004
Tris				6.06		
Tryptone	1.00	15.0				
Yeast Extract	0.500					
ZnSO_4_.7H_2_0				144 × 10^−6^		180 × 10^−6^
**Gelling agents**						
Agar	15.0	15.0	15.0	20.0	15.0	18.0
Gellan Gum	30.0	30.0	30.0	30.0	30.0	30.0

Products are given in gram per litre of distilled water. Gelling agents are not added in case of liquid media. Products marked in grey were filter sterilized before being added to autoclaved media in order to prevent caramelization of the sugars.

The complex media that were selected contain carbon and amino acids sources with unknown composition (tryptone, yeast extract, soytone, casein, starch), as well as high amounts of nutrients. This produces a rich environment for all bacteria to grow, although very different from the habitat inside plant tissues. The minimal media, on the other hand, contain an exactly known composition of nutrients and sugars, however in lower concentrations than complex media. In literature, higher numbers of bacteria have been shown to grow on complex media compared with minimal media (Jensen and Hammer, 1993; Hottes *et al*., 2004; Majzlik *et al*., 2011). Yet, in certain cases, some slow-growing bacterial strains were only found on minimal media after a prolonged growth period with less dominant strains to compete against (Connon and Giovannoni, 2002; Alain and Querellou, 2009). In this work, the numbers of bacteria were highest when grown on 1/10 diluted 869 medium for both root and shoot tissue (*P* < 0.05). The main difference between 869 medium and the other complex media (TSA and Casein-Starch) is the presence of glucose and yeast extract, which are not present in TSA and Casein-Starch media, and might be responsible for the increased growth on 869 medium. All bacterial nutrient media were tested with agar and gellan gum, but no notable differences were observed between both gelling agents. However, gellan gum did cause insufficient congelation in the M3 minimal medium. This might be due to the fact that gellan gum requires sufficient salts to congeal properly (Tamaki *et al*., 2005), and M3 contains a very low amount of salts. Therefore, no results are available for M3 medium with gellan gum.

Because of the obvious advantages of using 1/10 diluted 869 medium for the isolation of cultivable endophytic bacteria, this medium was used for the next steps of the study. No clear differences were evident between agar and gellan gum; therefore, both agents were tested in the remaining experiments.

#### Addition of plant extract to the nutrient media

**Fig 2:**
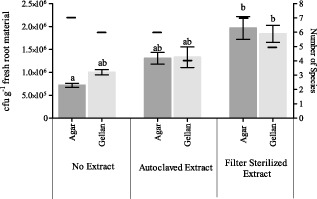
Comparison of the number of bacteria (in colony-forming units (cfu) per gram of fresh root material) growing on 1/10 diluted 869 medium with both agar and gellan gum as gelling agent and with autoclaved or filter sterilized plant extract (average of three technical repeats). The number of species that were visually distinguishable are given on the secondary y-axis. Bars with different letters are significantly different from each other (*P* < 0.05).

Each isolate was plated on six different variants of 1/10 diluted 869 medium: (i) no plant extract added, with agar; (ii) no plant extract added, with gellan gum; (iii) plant extract added before autoclaving, with agar; (iv) plant extract added before autoclaving, with gellan gum; (v) filter sterilized plant extract added after autoclaving, with agar; and (vi) filter sterilized plant extract added after autoclaving, with gellan gum. The 1/10 diluted 869 medium without plant extract yielded the lowest numbers of endophytes (7.1 × 10^5^ cfu g^−1^ for agar and 1.0 × 10^6^ for gellan gum) of all three conditions (Fig. 2). Adding filter sterilized plant extract significantly increased the numbers of cultivable endophytic bacteria (*P* < 0.05). No difference was observed when comparing the numbers of visually distinguishable strains over the different conditions.

#### Genotypic characterization of endophytes on different nutrient media

**Fig 3:**
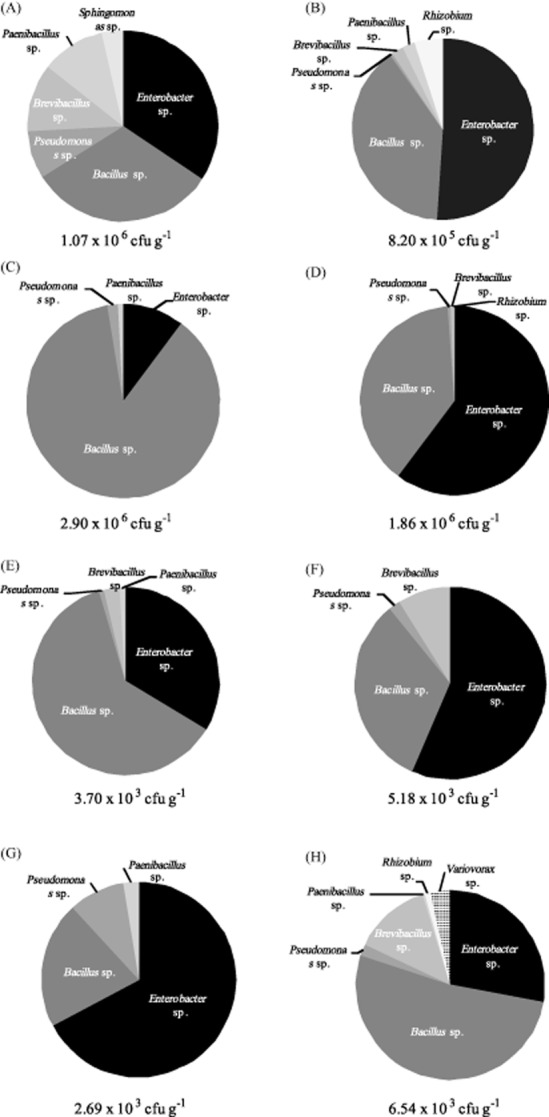
Genotypic characterization of the cultivable bacterial population of *Cucurbita pepo* grown on different adaptations of 1/10 diluted 869 medium with agar or gellan gum as gelling agent and with or without filter sterilized plant extract.A. Root endophytes on 1/10 diluted 869 medium with agar.B. Root endophytes on 1/10 diluted 869 medium with agar and root extract.C. Shoot endophytes on 1/10 diluted 869 medium with agar.D. Shoot endophytes on 1/10 diluted 869 medium with agar and shoot extract.E. Root endophytes on 1/10 diluted 869 medium with gellan gum.F. Root endophytes on 1/10 diluted 869 medium with gellan gum and root extract.G. Shoot endophytes on 1/10 diluted 869 medium with gellan gum.H. Shoot endophytes on 1/10 diluted 869 medium with gellan gum and shoot extract.

**Fig 4:**
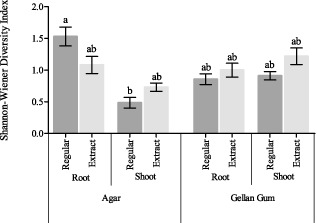
Comparison of the Shannon-Wiener diversity index for the cultivable population of *Cucurbita pepo* grown on different variations of 1/10 diluted 869 medium with agar or gellan gum as a gelling agent, from root and shoot material and with or without the addition of filter sterilized plant extract (averages of three technical repeats). Bars with different letters are significantly different from each other (*P* < 0.05).

In a final isolation, root and shoot isolates were spread on four different variants of 1/10 diluted 869 medium in triplicate: (i) without plant extract, with agar; (ii) with filter sterilized plant extract, with agar; (iii) without plant extract, with gellan gum; and (iv) with filter sterilized plant extract, with gellan gum. All cultivable bacterial strains were analysed genotypically so as to characterize the cultivable bacterial populations as a function of growth medium (Fig. 3). In total eight genera were detected: *Enterobacter* sp., *Bacillus* sp., *Pseudomonas* sp., *Brevibacillus* sp., *Paenibacillus* sp., *Sphingomonas* sp., *Rhizobium* sp. and *Variovorax* sp. The cultivable populations did not show much variation across all growth conditions, with *Enterobacter* sp., *Bacillus* sp. and *Pseudomonas* sp. typically being the dominant groups. The average Shannon-Wiener diversity index was calculated for all growth conditions (Fig. 4) as to estimate the diversity of cultivable strains. When comparing the diversity in conditions with agar or gellan gum as a gelling agent, both increases and decreases in diversity were noticed; therefore, no conclusive effect of the gelling agent could be observed. In general, an increasing trend was observed when plant extract was added to the medium (except for the isolation of endophytes from root tissue on agar plates).

### Optimization of growth capacity when regrown after isolation

#### Addition of plant extract to medium for regrowing endophytes from glycerol stock

After isolation, endophytes are typically stored in a glycerol solution (15%_w_) at −80°C, with the assumption that activity is conserved under these conditions for later experiments focused on plant growth-promoting capacities and tolerance/degradation experiments or for inoculation. However, to conduct these types of experiments, endophytes need to be effectively regrown from the glycerol stock and this often produces problems since the number of viable cultivable cells can decrease upon storage in glycerol (Spira *et al*., 2011; Kim *et al*., 2013). In the current study, although these bacteria were growing on 1/10 diluted rich 869 medium during the original isolation, some strains do not show any viability when inoculated into the same, yet undiluted, medium from glycerol stock. We speculate that this may be due to the fact that during the original isolation, residual plant extract is present; however, upon regrowth from the glycerol stock, no plant extract is present. To address this possibility, several bacterial strains that showed little regrowth from glycerol stock were inoculated on both 869 and 284 medium with and without plant extract. These bacteria originated from *A. thaliana* and *C. pumila* (Table 2).

**Table 2:** Endophytes regrown from glycerol stock

Seed endophytes *A. thaliana*	Total number	869	869+ Extract	284	284+ Extract
*Bacillus*	4	4	4	0	2
*Microbacterium*	5	5	5	0	4
*Paenibacillus*	6	6	6	0	5
*Staphylococcus*	5	5	5	0	2

Stem endophytes *A. thaliana*	Total number	869	869+ Extract	284	284+ Extract
*Azospirillum*	1	0	1	0	0
*Eubacterium*	1	1	1	0	0
*Fluviicola*	3	3	3	0	0
*Kaistia*	1	0	1	0	0
*Nevskia*	1	0	0	0	0
*Paenibacillus*	1	1	1	0	0
*Pedobacter*	1	1	1	0	1
*Pelomonas*	2	0	2	0	0
*Pseudomonas*	1	0	0	0	0
*Sediminibacterium*	2	1	2	0	0
*Sphingomonas*	2	0	2	0	0
*Staphylococcus*	2	1	2	0	2
*Variovorax*	2	1	2	0	2

Leaf endophytes *A. thaliana*	Total number	869	869+ Extract	284	284+ Extract
*Acidovorax*	3	3	3	0	2
*Bacillus*	2	1	2	0	0
*Brevundimonas*	2	0	1	0	0
*Exiguobacterium*	2	2	2	0	2
*Fluviicola*	7	5	7	0	2
*Microbacterium*	1	0	0	0	0
*Pelomonas*	20	2	17	0	3
*Pseudomonas*	2	0	2	0	0
*Rhizobium*	4	1	2	0	0
*Rhodococcus*	1	0	1	0	0
*Sediminibacterium*	2	0	2	0	0
*Sinorhizobium*	1	1	1	0	0
*Variovorax*	1	0	1	0	1

Seed endophytes *C. pumila*	Total number	869	869+ Extract	284	284+ Extract
*Bacillus*	3	3	3	1	2
*Brachyobacterium*	5	4	5	3	5
*Curtobacterium*	4	4	4	1	2
*Caulobacter*	1	0	1	0	0
*Microbacterium*	1	1	1	0	0
*Staphylococcus*	6	6	6	3	5
*Sphingomonas*	12	8	12	7	10

Endophytes were isolated from seed, stem and leaf tissue of *Arabidopsis thaliana* and seed tissue of *Crotalaria pumila*. The bacterial glycerol suspension was spread on 869 and 284 medium with and without plant extract with agar as a gelling agent. ‘Total number’ depicts the number of bacteria that is attempted to be grown from glycerol stock; the other columns depict the number of bacteria that grew on the plates.

**Fig 5:**
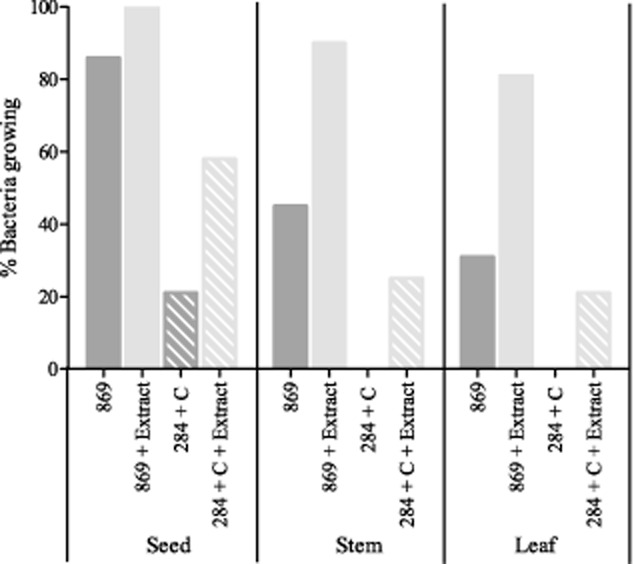
Percentage of bacteria originally isolated from seed, stem and root tissue of *Arabidopsis thaliana* and seeds of *Crotalaria pumila* that show growth when regrown out of glycerol stock onto 1/10 diluted 869 medium with and without plant extract and 284+ C medium with and without plant extract.

In general, growing the bacteria on the rich 869 medium showed less problems than growing bacteria on the selective 284 medium. But since certain experiments/tests require bacteria to be grown on 284 medium, both options were evaluated. Figure 5 contains the summary of the growth from endophytes isolated from different plant tissues. Increases of growth were observed for endophytes from all plant tissues when plant extract was added to 869 or 284 medium. As such, it appears clear that some endophytes are unable to adapt to new growth media and conditions without the presence of certain, unidentified, compounds present in plant tissue. Although we only tested and proved this method in the case of plant endophytes, the same concept might be useful in case of other microbiological applications. In general, adding a sterilized extract of the specific tissue of the species from which bacteria were isolated might increase the regrowth of bacteria after storage since the adaptation to new media might be facilitated.

### Conclusions

For isolation of endophytes from plant tissue, 1/10 diluted 869 (rich) medium proved to deliver the highest numbers of cultivable endophytes, as well as the highest diversity. When comparing agar and gellan gum as gelling agents, no obvious differences were noticed, although gellan gum was less suitable for application in selective media in which little amounts of salts are present since solidification of the medium is compromised. During the isolation process, the addition of plant extract significantly increased the numbers of isolated bacteria (*P* < 0.05), but had little effect on the diversity of the cultivable endophytes.

Bacteria that were formerly isolated and stored, however, showed improved growth potential when filter sterilized plant extract was added to either rich 869 medium or minimal 284 medium.

## Experimental procedures

### Preparation of bacterial growth media

Media were prepared according to Table 1. All media were adjusted to pH 7 with either HCl or NaOH before autoclaving.

To acquire plant extract, 1 g of root or shoot source tissue of the plant species from which bacterial isolation was desired was crushed with 5 ml of sterile 10 mM MgSO_4_. The extract was added either before autoclaving or through filter sterilization (Minisart single use filter unit 0.2 μm, Sartorius Stedim Biotech, Germany) to the medium after autoclaving. Both methods were evaluated since heat and pressure associated with autoclaving could cause degradation of essential compounds present in the plant extract.

### Growing plants

Seeds from *C. pepo* (Johnny's Selected Seeds, Winslow, ME, USA) were incubated on humid paper towels at 30°C for 3 days to germinate. The seedlings were transferred to plastic pots (750 ml) containing vermiculite and were saturated daily with one-fourth Hoagland nutrient solution. The pots were maintained in a greenhouse (humidity 60%; day night cycle: day 7.00–22.00; temperature: day 23°C, night 18°C; light intensity 300 W m^−2^) for 21 days prior to harvest.

### Surface sterilization of plant tissues

To surface sterilize plant tissues, vermiculite was removed from the roots with tap water. Plant mass was determined prior to separating roots and shoots. The individual plant tissues were incubated for 1 min in 1% NaOCl for external sterilization. Subsequently, plant tissues were rinsed three times in sterile distilled water prior to drying on sterilized filter paper. One hundred microlitre of the third rinsate was plated on undiluted rich 869 medium to confirm sterilization.

### Isolation of cultivable bacterial strains

To isolate cultivable bacteria, surface sterilized root and shoot tissue of three plants were separated and transferred to mortars with 5 ml sterile 10 mM MgSO_4_ to compose a mixed sample. The plant tissues were separately crushed and serial dilutions were prepared (0, 10^−1^, 10^−2^, 10^−3^, 10^−4^). One hundred microlitre of each dilution was applied to the Petri dishes containing different media (Table 1); all Petri dishes were established in triplicate. The Petri dishes were incubated at 30°C for 4 days after which the cfu per gram of fresh plant tissue was determined; the averages and standard errors were calculated for each treatment. The colonies were then purified and 584 strains were stored at −80°C in glycerol (15%_w_ glycerol, 0.85%_w_ NaCl).

### Genotypic characterization of cultivable isolated bacteria

Each isolated strain was subjected to DNA isolation using the Qiagen DNeasy Blood and Tissue Kit (Qiagen, Venlo, the Netherlands). A Nanodrop ND-1000 Spectrophotometer (Isogen Life Science, De Meern, the Netherlands) was used to analyse the quality and quantity of the extracted DNA. For the amplification of the 16S rDNA, aliquots of the extracted DNA were used directly. The universal 1392R primer (5-ACGGGCGGTGTGTGTRC-3) was combined with the bacteria-specific 26F primer (5-AGAGTTTGATCCTGGCTCAG-3) for prokaryotic 16S rDNA amplification as described by Weyens and colleagues (2009). The 16S rDNA products were digested and separated by gel electrophoresis (90 V, 2 h) with a 1.5% agarose, gelred nucleic acid stained gel and were then visualized under ultraviolet illumination. The 16S fingerprint profiles were analysed and from the isolates showing the same band pattern, at least one representative strain of 33 different band patterns was selected for 16S rDNA sequencing. Partial 16S rDNA sequences were obtained from Magrogen (Amsterdam, the Netherlands). Consensus sequences were compiled with the Staden Package after which sequence match from the Ribosome Database Project was used for species identification.

After identification of the cultivable population, the average Shannon-Wiener diversity index was calculated from the treatment groups so as to compare the efficacy of the different growth conditions (Spellerberg and Fedor, 2003; Keylock, 2005).

### Regrowth of previously isolated endophytes

The regrowth of previously isolated endophytes was tested on four different media types: (i) undiluted rich 869 medium with agar, without plant extract; (ii) undiluted rich 869 medium with agar, with plant extract; (iii) selective 284 medium with agar, without plant extract; and (iv) selective 284 medium with agar, with plant extract. Undiluted 869 medium was used since the strains were purified and no risk of different species overgrowing each other existed. Ninety-eight bacterial strains of 21 phyla isolated from *A. thaliana* and 32 strains of 7 phyla isolated from *C. pumila* that showed constraints growing from glycerol stock were tested. The bacteria were directly transferred from the glycerol stock to the media with a sterilized inoculation loop. The plates were incubated for 5 days at 30°C before being checked for growth. If bacterial colonies were present, the regrowth was considered positive, and the lack of colonies was considered negative.

### Statistical analysis

All averages and standard errors were calculated from three replicates from a mixed sample of plant material from three plants. The statistical differences were analysed by using a Kruskal–Wallis non-parametric test and a Dunn Test using R (R Foundation for Statistical Computing, Vienna, Austria).
